# Respiratory Motion Sensor Measuring Capacitance Constructed across Skin in Daily Activities

**DOI:** 10.3390/mi9110543

**Published:** 2018-10-24

**Authors:** Makie Terazawa, Momoko Karita, Shinya Kumagai, Minoru Sasaki

**Affiliations:** 1 Department of Advanced Science and Technology, Toyota Technological Institute, Nagoya 468-8511, Japan; sd11061@toyota-ti.ac.jp (M.T.); sd12021@toyota-ti.ac.jp (M.K.); 2 Department of Electrical and Electronic Engineering, Faculty of Science and Technology, Meijo University, Nagoya 468-8502, Japan; skumagai@meijo-u.ac.jp

**Keywords:** respiratory sensor, wearable sensor, capacitance constructed across skin

## Abstract

In this work, a respiratory sensor is studied, measuring the capacitance constructed by attached electrodes on the abdomen. Based on previous findings, that skin thickness changes caused by respiration provides the signal, the fitting condition of the electrode on the skin is stabilized using a 7-μm-thick dressing film. This film can be comfortably worn for a long time, while maintaining the electrode’s position on the skin. This stabilized setup enables the detection of, not only respiration, as the cyclic capacitance change, but also of minute body volume changes over the daytime, as a change in the base line indicates the quality of the sensor signal. For this demonstration, the respiration signal is measured during the daily activity of exercise and 6-min walks.

## 1. Introduction

Recently, sensors suitable for continuous monitoring of a patient’s condition have gained attention from the medical and healthcare fields. As clear examples, diabetic patients are required to measure their blood glucose levels [1], and some patients after an operation are required to monitor signals. In the future, the daily monitoring of a subject’s health and activity will be considered in order to detect a change in their physical condition and for early detection of diseases. Presently, there are many wearable sensors on the market. However, the majority are products for athletes, usually measuring heart rate, temperature and accelerations [2,3,4]. Even in sensors for healthcare applications [5,6], they rarely include a respiration sensor, as it is not established.

Respiration is an important vital sign; however, it is said to be the most neglected one [7]. Since respiration is consciously controllable, a subject natural becomes important for obtaining a good signal. The spirometer, a standard respiration sensor, uses a flowmeter connected to a mouthpiece, or a mask with a tube. The air volume of the expiration is measured. The subjects are tethered to the equipment and such a setup restricts a subject’s natural activity [8]. The belt-type strain sensor using piezoresistive fabric is studied because it does not mask the mouth [9]. However, the inner garment surrounding the body inevitably causes stress based on the sensing principle. This stress is a significant load for people with weak health.

New sensing methods are being studied for realizing no stress to the subject. A t-shirt made of metal-glass-polymer fibers has been used as the spiral antenna, which changes the resonant radio frequency due to the subject’s body volume change caused by respiration [10]. The mask-type moisture sensor, made of hygroscopic paper, is used to measure patterns and respiratory rate [11]. Detecting the breathing sound from inside the body is one approach and its challenge is the recognition of respiratory sound patterns [12]. The algorithm for extracting the respiratory rate from waveforms acquired by a non-invasive photoplethysmogram has been discussed [13]. Multimodal patch sensors (combining three-axis accelerometers, three-axis gyroscopes, and three-axis magnetometers) placed on the body are used to measure respiratory frequency [14]. Some products are considered for measuring the breathing patterns from the signal frequency and the waveform of the nine-axis motion tracking devices, together with the sensor fusion algorithms, although the principle is kept in secret [15,16,17]. Even when the software algorithm is used, the original sensor signal quality becomes important for estimation. We found that measuring the capacitance [18] between two electrodes on the skin yields a promising respiration signal [19], as the capacitance is built across the gap without strain to the body. This capacitive sensor has the possibility of estimating the air volume, because the skin stress and air volume of expiration can be connected to information about the subject’s body size. Thus, checking the signal quality has become a research interest. In a previous study [19], sensing electrodes were fixed inside a T-shirt, so the relative movement between the T-shirt and the body causes the large noise of the signal jump, making sensing performance unclear.

In this study, electrodes are fitted on the skin in order to clarify the signal quality [20,21], and the sensor signal is measured during the possible motions of daily activities.

## 2. Method for Fitting Electrodes on the Skin

**Figure 1:**
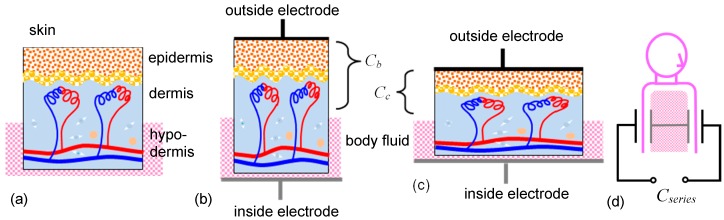
(**a**) Skin structure. Model of the electrodes constructing the capacitance across the skin under (**b**) exhalation and (**c**) inhalation conditions. (**d**) The two capacitances are considered to connect in series inside the body and are measured by the outside circuit.

Figure 1 shows the skin model, based on a previous study [22], confirming the signal tendency under a variety of conditions with repeatability. The capacitance is constructed between one electrode attached on the skin and another as the electrolytes inside, which is the conductive body fluid. The interior of the human body is considered to be a conductive electrolytic solution, and its resistance will be small. Figure 1b shows the conditions at exhalation. The body volume decreases and the skin contracts, increasing its thickness. The gap between the two electrodes will increase, and as such the capacitance decreases. Figure 1c shows the conditions at inhalation. The body volume increases, decreasing skin thickness. The gap between the two electrodes will decrease, and so the capacitance increases. The sensor measures the series capacitance made by the two capacitances beneath two outside electrodes, as shown in Figure 1d. Since the capacitance is constructed in the area beneath the skin, the signal has information from inside the body, which is an advantage of this method.

In order to obtain the signal without the noise caused by the relative movement between the electrode and the body, the electrode was attached to the skin, not inside the clothing. Three attaching tapes (listed in Table 1) were tested. Sports tape (3 M, Multipore Sports Lite Elastic Tape, Tokyo, Japan) suffers from drift noise; silicone gel sheet for covering a scab (Harasawa Pharmaceutical Co. Ltd., Tokyo, Japan) was tested (which sticks to the skin stably, but the signal magnitude decreases because the sheet is thick (2 mm)), but this caused the outside electrode to be far from the skin. Dressing film (Kyowa limited, Airwall™, Osaka, Japan) stabilizes the signal, maintaining the signal magnitude, because it is 7 μm-thick. This film has tiny holes for breathability, avoiding the problem of the electrode becoming wet due to sweat. Sweat is conductive and causes leaking of the current’s path on the skin surface. Capacitance measurements need dry skin conditions. The film maintains comfort and the electrode positions and conditions are maintained for a long time.

**Figure 2:**
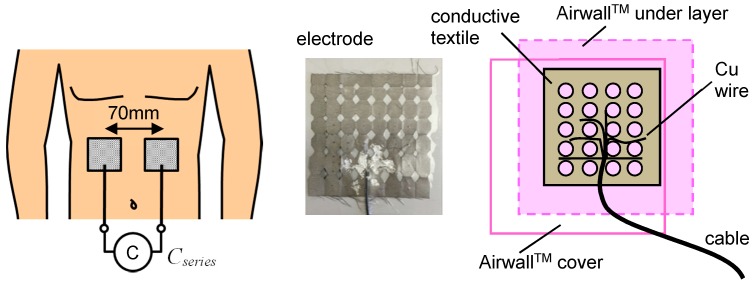
Typical electrodes attached to the skin; conductive textiles are used.

Figure 2 shows a schematic drawing of the electrodes and the measurement setup. Two electrodes are attached to the abdomen. Airwall™ film is very thin and transparent and the holes in the conductive textile are used for fixing the cover film. The lateral distance between the two electrode centers was 70 mm. The longitudinal position was the middle between the navel and the epigastric fossa. The subject does not touch the ground electrode. The sensing electrodes are not covered by the grounded conductive film. According to previous results [22], this setup is considered to make the parasitic capacitance smaller.

## 3. Results

### 3.1. Signal under Gentle Conditions in Daytime

**Figure 3:**
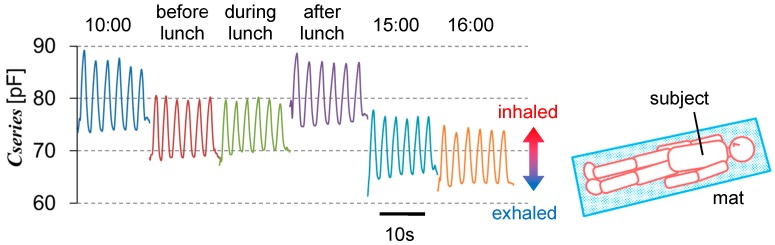
Typical daily capacitance change, including respiration. Since the posture generates the capacitance change, it is stabilized by lying on a mat.

Figure 3 shows six short-period signals for about 15 s. Time passes hours between the measurement time points. The capacitance between two 40 × 40 mm^2^ electrodes was measured using an impedance analyzer (Hioki IM3570, Nagano, Japan) at a frequency of 400 kHz. The subject lay on a mat in the same posture each time, as the signal changed depending on the posture. In all signals, a cyclic change with an amplitude of about 10 pF was observed; this was the respiration signal. The signal amplitude was more than 10% of the total capacitance value. The baseline decreased from 10:00 a.m. to before lunch, and from after lunch to 4:00 p.m. These periods corresponded to fasting. On the other hand, the baseline increased from before to after lunch. This period corresponded to eating. The body volume increased, decreasing the skin thickness. The capacitance was able to detect this small change. Including other experiments, the increasing ratio of the baseline at satiety against that at fasting was 14–22%. Considering that the physical skin thickness was about 1.5–4 mm, the change in the capacitance gap was estimated to be 0.1–1 mm. From the body structure, body expansion will direct to the abdomen since the backbone does not expands. The abdominal skin is a good position for the measurement because the body expansion effect gathers. The sensor electrode size can be smaller compared to those of belt-type strain sensors.

Each measurement time was about 15 s, taking about 830 data points. Since such data show a smooth curve, the capacitance measurement itself was stable. Their peak and valley values are statistically evaluated in Table 2. There are six peak and five valley values for each time point. The data waveforms were distorted at the start and end. The reason for this was that the switch-on/off operation were done by hand. These valley values are not included in the list. The peak capacitance values have a standard deviation of about 1% or less against the average. This may include fluctuation of natural respiration, even though the subject intends to maintain the same level of respiration.

### 3.2. Singals during Twisting/Bending of the Upper Body

**Figure 4:**
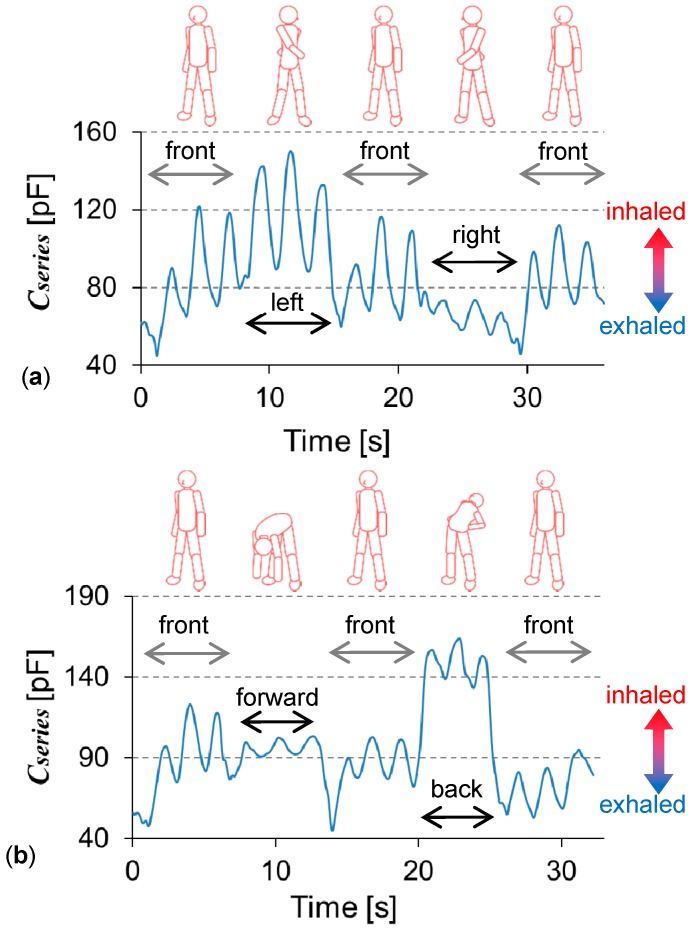
Abdomen capacitance signals during exercise. (**a**) Twisting the upper body left and right. (**b**) Bending the upper body forward and backward.

Figure 4a shows the capacitance change when the upper body was twisted about 90° to the left and right. The capacitance between two 50 × 50 mm^2^ stretchable textile electrodes was measured. Maintaining each posture as much as possible, respiration was measured three times, showing the cyclic capacitance change. The baseline shifts corresponded to the posture. The signal difference between twisting to the left and right was explained by the difference in the electrode conditions attached to the skin. The left electrode was fixed using the underlying dressing film, as well as the cover film. The right one was fixed using only the cover film. Twisting to the right, the right electrode tended to have a gap between the underlying skin; as such, the respiration signal decreased. When the electrodes were fixed on the garment, and not the skin, the capacitance signal changed much more, since exercise changed the relative position between the skin and the electrode. In Figure 4a, the body faced forward three times. The capacitance baselines are nearly the same for each time. The baseline seemed to respond following the body’s twisting movement. From the peaks, even with the baseline shift, the respiratory rate could be obtained. Figure 4b shows the capacitance change when the upper body bent about 90° forward and back (to the subject’s limit). Again, the respiration was measured three times in each posture. When the upper body bent, the changing ratio from the baseline became smaller than that under the normal conditions facing the front. When bending forward, skin will contract, increasing its thickness; as such the capacitance decreases. When bending backward, skin will expand, decreasing its thickness, and so capacitance increases. This is consistent with the model shown in Figure 1. The posture with skin sagging or stretching decreased the changing ratios generated by respiration. Table 3 and Table 4 lists the respiratory rates calculated from the central two periods in the three respiration cycles. The change in the respiratory rate against posture was smaller in twisting than in bending motions. This may relate to a change in the ease of respiration, depending on the posture.

### 3.3. Signals during Walking for Six Minutes

**Figure 5:**
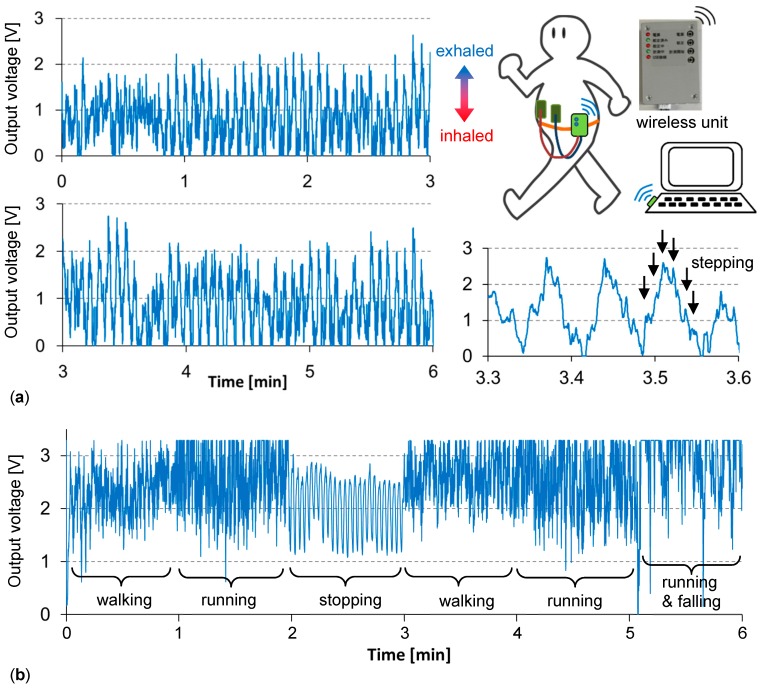
Abdomen capacitance signals measured by the wireless unit (**a**) during gentle walking and (**b**) during harder walking for 6 min.

Figure 5a shows the signal during gentle walking. A 6-min walk is the standard test in medical diagnoses checking the condition of the lungs. Here, the capacitance measuring circuit with a Bluetooth wireless unit (44 × 64 × 26 mm^3^) was used to allow “free walking”, as shown in Figure 5. This circuit works with two AAA batteries. To use this unit, the electrode size was designed and matched, proving the model validity in Figure 1. One electrode was 35 × 35 mm^2^, on the pit of the stomach, and another electrode was 70 × 70 cm^2^, on the abdomen, and was used for increasing the capacitance value. Due to the circuit specification used, a larger capacitance gave a smaller output voltage. As the amplification ratio was tuned and fixed monitoring of the signal occurred for 5 s after the switch was turned on, the signal ranged based on the time. The periodic change, having a magnitude of about 2 V, was the respiration signal; the respiratory rate could be obtained. The respiratory rate was stable at 15 cycle/min during the period from 1–6 min. This constant respiratory rate indicated the gentleness of the exercise. The respiration depth was related to the magnitude of the cyclic signal. The magnified waveform, from 3.3 to 3.6 min, showed smaller peaks, corresponding to the stepping motion, which influenced the skin condition beneath the sensing electrodes. Figure 5b shows the signal with more hard motions during 6-min. For each 1-min period, the subject performed the following motions many times: Stopping, walking, running, and running with falling. When stopped, the respiratory rate was about 24 cycle/min. During running, it increased to 26–27 cycle/min. This increase can be attributed to a subject’s metabolism. For each motion, the waveform tendency changed. The respiration signal was clearest at stopping, and noisiest at falling. Thus, the noise mainly showed body motion, rather than respiration. The baseline drift may relate to the posture change. When a weak-health subject walks, there is the possibility of falling or staggering. These relatively larger body movements were repeatedly simulated in order to obtain certain results.

## 4. Discussion

The advantage of the proposed sensor is that the sensor signal is obtained inside the clothes, directly from the skin of the body. Other previous sensors acquire signals from elements placed outside of a subject’s body. When the clothing fits to the body, the signal shows a subject’s information from the strain. Since the clothing and the body can slip over each other, the sensor signal will suffer from drift or offset. As for the proposed sensor, when the electrode was fit stably on the skin, it was found that it was possible to detect minute body changes caused by eating a meal (as the baseline shift). Thus, the capacitance signal was essentially highly sensitive for body conditions. As body movements, other than respiration, mix as noise, one future direction of the technique is separating respiration from other body movements. Body movement is also important information for monitoring people with poor health.

The next aim will be detecting changes in respiration in response to a subject’s metabolism. For example, detecting an increase in respiratory rate or magnitude in response to exercise. An algorithm extracting the respiration information from an original sensor signal with noise will be required. In fact, the described sensor can detect movements relating to skin displacement. When the electrode was on a leg or arm, its movement was detected. The capacitance signal shows the leg or arm movement, not respiration [22]. Because the sensor electrode area was relatively small, many electrodes can be attached to different locations of the body to detect, not only respiration, but also body movement. This will keep the hardware setup the same, including the capacitance measurement circuit, by simply increasing the number of channels. For the sensor hardware, electrode protection against sweat will eventually be necessary.

## 5. Conclusions

A novel respiratory sensor was studied, measuring the capacitance constructed across the skin. The skin contracts and expands due to respiration. This modulated the effective skin thickness of the constructed capacitance. When the electrode was fit stably on the skin, it was found that it was possible to detect minute body changes caused by the eating of a meal (as the baseline shift). The capacitance signal was considered to be highly sensitive. Respiration was detected during daily activity. Measurement during a 6-min walk was demonstrated. Although body movements other than respiration mix as noise, the respiration signal was observed.

## Figures and Tables

**Table 1:** List of tapes tested for fixing the electrodes.

-	Sports Tape	Silicone Gel	Dressing Film
Original use	For taping in sports	For artificial scabs	For fixing the needle of a drip infusion
Specification	0.4-mm-thick tape used for under-layer and cover.	2-mm-thick plate used for under-layer. Cover is sports tape.	7-μm -thick films used for under-layer and cover.
Setup for fixing electrode	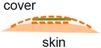	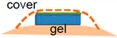	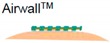
Signal evaluation	Drift is large for long time measurement.	Signal is stable but its magnitude decreases.	Signal is stable and large.
Judgement	bad	bad	good

**Table 2:** List of statistical evaluations of the peak and valley values in Figure 3.

-	Peak			Valley		
Timing	Average (pF)	Standard Deviation (pF)	Ratio	Average (pF)	Standard Deviation (pF)	Ratio
10:00 a.m.	87.15	1.170	0.0134	73.83	0.201	0.0027
Before lunch	80.08	0.372	0.0046	68.67	0.282	0.0041
During lunch	79.66	0.394	0.0049	69.81	0.319	0.0046
After lunch	87.16	0.669	0.0077	75.02	0.321	0.0043
3:00 p.m.	76.65	0.523	0.0068	65.25	0.422	0.0065
4:00 p.m.	74.10	0.439	0.0059	63.67	0.255	0.0040

**Table 3:** Average respiratory rate during exercise (shown in Figure 4). Twisting the upper body left and right.

Posture	Front	Left	Front	Right	Front
Period in Figure 4a (s)	2.43–6.89	9.49–14.15	16.76–21.06	23.57–27.98	30.51–34.64
Respiratory rate (cycle/min)	26.9	25.7	27.9	27.2	29.1

**Table 4:** Average respiratory rate during exercise (shown in Figure 4). Bending the upper body forward and backward.

Posture	Front	Forward	Front	Back	Front
Period in Figure 4b (s)	2.30–5.90	7.92–12.59	15.09–18.78	20.93–24.50	27.02–31.23
Respiratory rate (cycle/min)	33.3	25.7	32.5	33.7	28.5
